# Recombinant hemagglutinin of swine H1N1 influenza virus expression in the insect cells: Formulation in Montanide ISA71 adjuvant and the potency studies

**DOI:** 10.22038/IJBMS.2021.57053.12716

**Published:** 2021-11

**Authors:** Sara Zahmati, Morteza Taghizadeh, Setareh Haghighat, Reza Jalalirad, Mehdi Mahdavi

**Affiliations:** 1 Department of Microbiology, Faculty of Advanced science and Technology, Tehran Medical sciences Islamic Azad University, Tehran, Iran; 2 Department of Research and Development, Razi Vaccine and Serum Research institute, Agricultural Research Education and Extension Organization; 3 (AREEO), Karaj, Iran; 4 Production and Research Complex, Pasteur Institute of Iran, Karaj, Iran; 5 Advanced Therapy Medicinal Product Department, Breast Cancer Research Center, Motamed Cancer Institute, Academic Center for Education,; 6 Culture and Research, Tehran, Iran; 7 Recombinant Vaccine Research Center, Tehran University of Medical Sciences, Tehran, Iran; 8 Immunotherapy Group, Pharmaceutical Sciences Research Center (PSRC), The Institute of Pharmaceutical Sciences (TIPS), Tehran University of; 9 Medical Sciences, Tehran, Iran; 10 Department of Immunology, Pasteur Institute of Iran, Tehran, Iran

**Keywords:** Adjuvant, H1N1 subtype, Hemagglutinin, Influenza, Recombinant vaccine

## Abstract

**Objective(s):**

Influenza is a highly contagious disease, which affects the respiratory system and seasonal influenza is common throughout the world. Influenza vaccination is an effective way to reduce the risk of death and hospitalization. This study aims at the expression of swine recombinant hemagglutinin protein in the baculovirus expression system and it offers a comparison of the immunologic parameters with the commercial vaccine.

**Materials and Methods:**

The HA gene from the swine H1N1 strain of the Influenza virus was cloned into the Bac-To-Bac expression system in pFastBAC HTA vector and was transformed into *Escherichia coli* TOP10 strain. After the confirmation, the vector was transfected into the SF9 insect cell line. The recombinant HA was evaluated by SDS-PAGE and western blot. After formulation in Montanide ISA71 adjuvant, the immunization test was performed comparatively with Alum adjuvant, commercial vaccine in four groups of BALB/c mice, of which one group was control without any vaccination. Two weeks after the last immunization, the antibody response was assessed with HI assay, and experimental mice were challenged with mouse-adapted Influenza A/PR8/34 (H1N1) virus through nasal inhalation.

**Results:**

The immunoassay results revealed that the candidate vaccine induced the antibody response as the commercial one did but it did not significantly reduce the mortality rate, body loss, and severe fever.

**Conclusion:**

To summarize, the results showed that the recombinant protein with the Montanide^TM^ ISA- 71 adjuvant developed a more appropriate level of immunity than Alum adjuvant, so it might be used as a safe and reliable vaccine against H1N1 virus for further research.

## Introduction

Influenza is an RNA virus from the *Orthomyxoviridae* family (1). Influenza is one of the most common diseases between humans, swine, and poultry. 10% of the world’s population is affected by influenza annually. Depending on the host’s immune system, ‌these strains cause various symptoms, from a common cold to severe complications and even death. It has the potential to change throughout the respiratory tract, but it mainly affects the lower respiratory tract (2). Additionally, it has resulted in hundreds of billions of dollars’ worth of damage to the world poultry industry. To give some pandemic examples, in Spain (1919–1918), Asia (1957), and Hong Kong (1968), 50 million, 1 million, and 20 million people lost their lives, respectively (3, 4). All these pandemics were caused by the emergence of a new strain of the virus in humans. Most of these variations occur when an influenza virus is transmitted to humans from other animal species or when a human receives new genes from an infectious virus in birds or pigs (5, 6). In April 2009, a new strain emerged that was a combination of avian influenza, pig, and human genes. This strain was first dubbed the H1N1/A, Swine influenza. It first appeared in Mexico, the United States, and several other countries, and consequently, the World Health Organization officially declared a pandemic on June 11, 2009 (7).

The most common human vaccine is the triple influenza vaccine, which contains inactive and purified material from three viral strains. For example, the vaccine contains material from two influenza types, namely, virus A and a type of influenza B (8, 9). TIV has no risk of transmitting the disease and it is safe (9). Vaccines might be ineffective for the *coming* year, as the virus is rapidly changing and new species are rapidly replacing previous species (10), especially in the case of hemagglutinin antigen (11). This protein function binds the virus to the susceptible cells and triggers infectivity (2). In fact, hemagglutinin plays three important roles during virus replication as it binds the virus to the cell surface receptors that contain sialic acid. Hemagglutinin is responsible for virus infiltration into the cell cytoplasm by binding the endocytosis membrane of the virus to the endosomal membrane. The result is that the viral nucleocapsid is eventually released into the cytoplasm.

Hemagglutinin is the most important target for a vaccine design, and influenza virus epidemics are the result of the antigenic structure (12). It seems that by blocking hemagglutinin through neutralizing antibodies, the virus can be eliminated soon (13).

The effect of antibodies on protection against influenza viruses depends on various factors, including age, infection, and antibody levels induced during previous infection (14). People at high risk of developing influenza include children, the elderly, and those with diabetes, heart disease, and a weakened immune system (15, 16). Increasing vaccine potency is therefore crucial, and as a critical component, adjuvants improve vaccine potency. Adjuvants refer to compounds that do not have immunogenic effects, but if administered with a specific antigen, they enhance specific immune responses against that antigen(17). Montanide, as water-in-oil and aluminum salts, has been widely used to stimulate immune responses in influenza vaccines. Aluminum salts stimulate the immune system by deploying the vaccine at the injection site, but Montanide acts by recruiting immune cells to the involved lymph nodes (18). Shokouhi *et al*. (2016) suggested that using recombinant triple tandem repeat m2 protein of influenza in complex with CpG alum adjuvant significantly promoted immunogenicity and viability (up to 60%) of BALB /C mice versus H1N1 A virus (19). Researchers developed MF59 adjuvant by methylglycol-chitosan that induced a strong humoral immune response when compared with normal MF59 and it had no side effects (20). Baculovirus as the gene carrier was introduced in 1983 and this system has been recognized as one of the best and most powerful eukaryotic systems for protein expression from then on. To date, a wide range of viral, fungal, plant, and animal genes have been expressed in insect cells through this method (21). The advantage of the eukaryotic expression system over the prokaryotic expression system is that the expressed protein undergoes post-translational modifications, such as formation of disulfide bonds, phosphorylation, oligomerization, and glycosylation. It can be assured that the synthetic protein is spatially similar to the 3D structure of the antigen at the surface of the virus (22). The vector, which was applied in this research, has a strong PUC replication origin and is capable of replicating in DH10. Also, replication in eukaryotic cells is made through the SV40 replication origin. Gene expression also happens under a strong polyhedrane promoter. This main feature of the vector is due to its mouse kappa immunoglobulin chain and the protein expressed by this vector is secreted out of the cell sequence, and it is not locked inside the cell.

This study is conducted by using the H1N1 strain prepared by Razi Vaccine and Serum Research Institute to develop and formulate a recombinant vaccine in Montanide^TM^ ISA-71 adjuvant for the first time in Iran. In this way, it could be a likely platform for future development of such novel influenza vaccines in Iran.

## Materials and Methods

***Amplification and recombinant bacmid construction***

**Figure 1:**
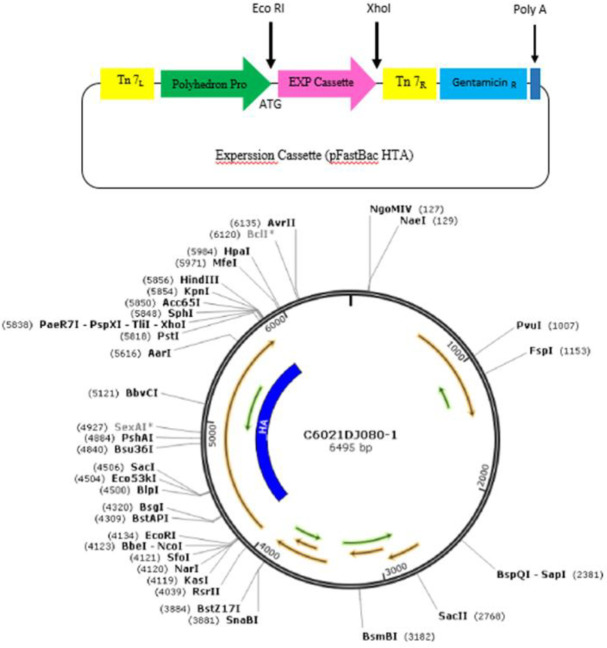
Expression cassette designed in plasmid pFAST Bac HTA, carrier of hemagglutinin H1N1 gene. The considered gene and vector were digested with XhoI and EcoRI restriction enzymes

In this study, the cDNA encoding HA derived from A/California/07/2009(H1N1) (Gene bank: NC_026433.1) was synthesized and inserted into the constructed cassette (Gene script Company, China) (Figure 1). Afterward, the target gene was cloned into donor vector, pFastBacHTA, between EcoRI and Xhol restriction site. The recombinant plasmid was confirmed through digestion and PCR. In both approaches, specific plasmid and gene primers were employed (Tables 1 & 2). According to Bac-to-Bac Expression System (Invitrogen), the recombinant plasmid was transformed into DH10Bac competent cells to construct recombinant baculovirus. The bacmid DNA contains M13 forward and reverse priming sites flanking the mini-att Tn7 site within the LacZ a-complementation region. Then, the transformed cells were cultured in Luria Bertani agar containing Gentamicin 7.5 µg/ml, Kanamycine 47 µg/ml, Tetracycline 10 µg/ml, Blue gal 100 µg/ml, and IPTG 40 µg/ml and were incubated for 48 hr at 37 °C. The white colonies were isolated and verified by PCR analysis through M13/pUC specific primers: 

M13/pUC Forward 5′-CCCAGTCACGACGTTGTAAAACG-3′, 

M13/pUC Reverse 5′-AGCGGATAACAATTTCACACAGG-3′.

***Insect cell culture and transfection***

Insect cell *Sf9*, derived from the *Spodoptera frugiperda* insect ovarian cell line, was obtained from the National Center for Genetic and Biological Resources of Iran (IBRC C10127). To propagate the cell lines, they were maintained at 26 °C in Grace’s insect medium (Gibco, Germany). After propagation, they were transfected with recombinant bacmid by electroporation protocols (60 voltage, 600 µF capacitance). The cells were infected with recombinant bacmid and were monitored daily for assessment of cytopathic effects (CPE). Recombinant baculoviruses were isolated from the flask’s supernatant (96 hr after culture and incubation at 27 °C) by centrifugation at 3000 ×g for 5 min. They were then transferred to a 4 °C refrigerator. 

***SDS PAGE and Western blotting***

Recombinant HA expression under the control of the polyhedron promoter was detected by SDS polyacrylamide gel electrophoresis followed by Western blot analysis. Infected sf9 monolayer cells were isolated from Grace medium by centrifugation at 3000 ×g for 5 min, and the cell pellets were washed with cold and protein-free PBS three times. Then, the pellet was resuspended in a lysis buffer (300 mM NaCl, 20 mM Tris–HCl (pH 8)), 1% Triton X-100, and 1X protease inhibitor cocktail (Sigma-Aldrich), and it was then placed on ice for 30 min. The cell lysate proteins were centrifuged at 12000 ×g for 5 min, then separated on 12% polyacrylamide gel SDS PAGE and transferred to nitrocellulose membranes for Western blotting. The Western paper was incubated in a blocking solution and shaken for 2 hr, then removed and washed twice with TBST 1X. Next, the HA protein reacted with primary antibody, Anti-His tag (diluted in blocking buffer (1:100)), and was shaken at room temperature for 1 hr. The paper was washed twice with TBST 1X (0.05% Tween 20 in PBS) for 15 and 10 min, respectively, and it was then incubated with HRP-conjugated IgG (Sigma Aldrich, USA) as a secondary antibody. After being washed 3 times (PBS/Tween 20), antibody binding was visualized by incubating the membrane with Sigma Fast diaminobenzidine (DAB) and metal enhancer (Sigma, Germany).

***Hemagglutination assay of the recombinant HA***

Following Killian (24), heparinized chicken blood was prepared and washed three times with PBS and the mixture was centrifuged at 1500 ×g for 7 min at 7 °C. In this way, 25 µl PBS was loaded to wells 1–2. Then, 50 µl of antigen was added to the first well of each row from a V-shaped 96-well plate, and serial dilution was performed in the successive wells and the final 50 µl was discarded from the last well. All wells were supplemented with 50 µl of 0.5% chick blood and incubated at room temperature. After 30 min, the titer of RBC agglutination was recorded. The last well that showed the complete hemagglutination was reported as one HA unit.

***Protein expression and purification***

Sf9 cells were inoculated with recombinant bacmid with MOI of 10 and incubated at 26 °C for three days. The flask’s cell suspension was collected by centrifugation at 10000 ×g for 20 min at 4 °C, and the supernatant was stored at -20 °C for the next step. The recombinant HA protein was purified by chromatography column Ni-NTA. Ni-NTA gel was transferred to a column and it was combined with ethanol 70%, and after shaking, the solution was discarded. Next, 4 ml lysis buffer (NaH_2_PO_4_ 0.86gr, NaCl 2.2 gr, DDW 125 ml, Imidazole 0.04 gr) was loaded on the column and discarded again. The supernatant was added to the column on ice and shaken for 2 hr at room temperature. At this point, the supernatant containing the recombinant protein passed through the column. The column was washed with washing buffer (NaH_2_PO_4_ 0.86gr, NaCl 2.2 gr, DDW 125 ml, Imidazole 0.08 gr) and then the recombinant protein was eluted with elution buffer (NaH2PO4 0.86gr, NaCl 2.2 gr, DDW 125 ml, Imidazole 2.1 gr). The purified protein was qualified and quantified by SDS-PAGE and Bradford assay, and it was then stored at 4 °C. Next, purified rHA antigen was formulated in Montanide ISA-71 adjuvant (SEPPIC, France) at the ratio of 40/60 using a homogenizer. The Lowry method was applied for purified volume measurement in 750 nm wavelength. The obtained result was calculated through the following equation where the resultant purified protein volume in 100 µl culture medium was determined 10.76 µg.

***Animals***

Seven-week-old female BALB/C mice (n=45) were obtained from Razi Vaccine and Serum Research Institute of Iran (Karaj, Alborz). The mice were housed at Animal Room (20-22 °C) of Razi Vaccine and Serum Research Institute**.**

***Experimental groups and immunization***

Experimental mice were divided into three groups (15 mice in each one). One group of mice was vaccinated with the commercial H1N1 influenza vaccine (Vaxigrip TetraTM 2018/2019 Sanofi Pasteur). The second group was immunized with the recombinant H1N1 protein formulated in MontanideTM ISA-71 adjuvant, and the last group acted as the negative control (the non-vaccinated group). Experimental mice were immunized with 5 μg of the vaccines, subcutaneously. Vaccination was performed on days 0 and 14. Two weeks after the second injection, the blood sample was collected, and serum was separated by centrifugation at 10000 ×g for10 min and stored at -20 °C for further experiments.

***ELISA of specific antibody responses***

The serum antibody levels of the immunized mice were detected by ELISA. ELISA plates (Greiner, Germany, Cat. No 655061) were coated with 100 μl of 5 µg/ml of recombinant protein in PBS and incubated overnight at 6 °C. The plates were then blocked by a washing buffer— containing BSA 1%—at 37 °C for 1 hr. In the next step, the plates were washed three times with washing buffer. Following this, 100 μl of two diluted sera, 1/10 and 1/100, samples were added to the wells and the plates with covering lid were incubated at room temperature for 90 min. The plates were then rinsed three times, and 100 μl of anti-mouse HRP-conjugate (Sigma, USA) was added to the wells, and the plates were incubated in a 37 °C incubator for 60 min. The plates were washed five times, and then 100 μl of TMB substrate was added to each well and kept in the dark. After about 30 min, 2N H_2_SO_4_ as stop solution was added to all wells and read immediately by an ELISA reader at 450 nm.

***Hemagglutination inhibition (HI) test***

Twenty-five µl PBS buffer was added to all wells of the 96-well plates except the first column. Instantly, 25 µl of inactivated serum was poured into each row’s first well, and serial dilution was performed just like before. Then, 25 µl of the standard virus (4 units) was added to all wells and incubated at room temperature for 60 min. After this period, 50 µl of 0.5% chicken blood was added to all wells and incubated at room temperature for 60 min. The wells with deposited cells were considered titers.

***Experimental virus challenge***

Two weeks after the last immunization, 4 female BALB/c mice from each group were infected intranasally. Accordingly, 5×10^5^ PFU of a mouse adapted influenza A/PR8/34(H1N1) virus in the Razi Vaccine and Serum Research Institute was used (23, 24). The mice for adaptation of influenza A/PR8/34 (H1N1) virus were obtained from Razi Vaccine and Serum Research Institute and used to determine the titer using the LD_50_ method (TCID50). Four mice in six groups were anesthetized with pentobarbital sodium and infected intranasally with 50×10^1^- 50×10^8^ TCID viruses per 50 μl. During this time, each group was kept in separate cages and under a biosafety level two animal house for 14 days, and after two weeks the final titer (10^5^) was determined as LD_50_. In this challenge, the daily weight of mice (in each cage) was measured separately with a calibrated digital balance (without the least stress on the mice). Additionally, the body temperature of the mice in each cage was measured twice a day with a thermometer without causing stress. The mortality rate was examined twice in the morning and at night.

***Statistical analysis***

After collecting the initial data from the experiments, the normality test was guided by the Kolmogorov-Smirnoff test. One-way ANOVA at 95% confidence level (*P*<0.05) was used to compare the experimental groups in terms of differences. *Post hoc* and Turkey tests were applied to compare the means between groups. Following Kaplan-Meier, the log-rank test was used for mortality rate analysis. The statistical analysis was performed in SPSS (version 15). 

The Ethics Committee at Islamic Azad Tehran Medical Sciences approved this project (approval ID: IR.IAU.PS.REC.1397.101).

## Results

***PCR amplification and cloning***

**Figure 2:**
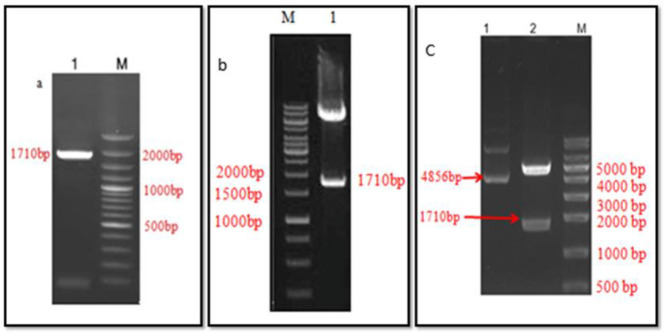
PCR amplification and cloning results. a. 1: HA gene amplified from H1N1 cDNA, 1710 bp, M: ladder. b. 1: PCR confirmation of vector cloned with HA gene, M: ladder. c. 1. Control vector (4856bp), 2, Digestion of recombinant pFAST Bac HTA with XhoI and EcoRI 4856 bp +1710 bp (HA gene)

To amplify the cDNA of the hemagglutinin gene, PCR was performed using the PFU enzyme. PFU enzyme corrects the made strands in a volume of about 100 µl. The interested bound (1710 bp) was visualized on 1% gel (Figure 2a).

Before ligation, the vector concentration was 50 ng/μl and the hemagglutinin gene was 34.7 ng/μl. The reaction was transformed into the bacterium and cultured on a selective medium to confirm hemagglutinin cloning. After purification of the plasmid and concentration determination, it was digested with two restriction enzymes, namely, Xhol and EcoRI (Jena Bioscience, Germany). It was then visualized on gel electrophoresis 1% (Figure 2b). Next, the transformation of DH10Bac cells was performed by recombinant pFastBacHTA, which act as donor plasmids. The white colonies containing the recombinant bacmid were selected and used for PCR validation by M13/ pUC forward and reverse primers (Figure 2c).

***Transfection of insect cells***

**Figure 3:**
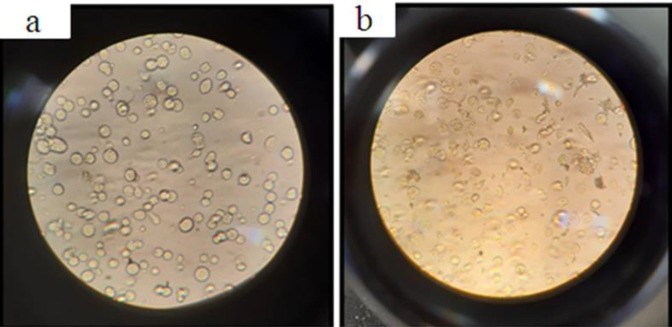
Cytopathic effects of Sf9 cells infected with recombinant bacmid. a: Sf9 cell before transfection. b: Sf9 cell transfected with recombinant bacmid after 8 days. Transfected cells are large, low density and destroyed walls

The cytopathic effects were visualized using inverter-microscopes at 24 hr post-infection with recombinant bacmid (Figure 3). As a result, the infected cell growth stopped and they were eventually detached from the monolayer and died. Cytopathic effects include ceasing the division, expanding the nucleus and cells, desiccating, and reducing density. 

***SDS PAGE and Western Blotting***

**Figure 4:**
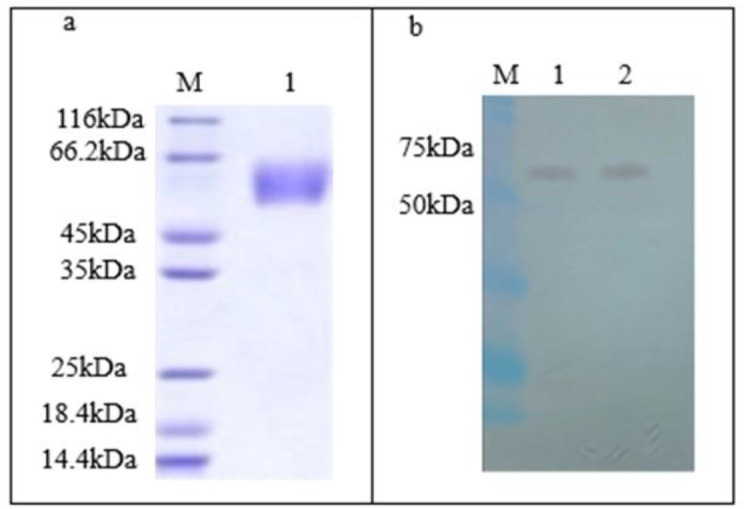
SDS-PAGE and western blot analysis. a. SDS-PAGE, M: protein ladder, 1: HA protein (66 kDa); b. Western blot, M: ladder, 1: positive control, 2: HA protein

For analysis of the expression of the recombinant protein, a baculovirus-infected insect cell containing the hemagglutinin gene was harvested after 72 hr. The protein of the cell lysate was electrophoresed on the 12% polyacrylamide gel SDS PAGE. As expected, the protein band with a molecular weight of approximately 66 kDa was detected (Figure 4a). Western blot analysis was performed with anti-His tag and monoclonal antibodies HRP conjugate, and it proved the detected bands’ specificity (a protein band between the ladder bands of 66-45 kDa equal to 64 kDa) (Figure 4b).

***HA assay***

**Figure 5:**
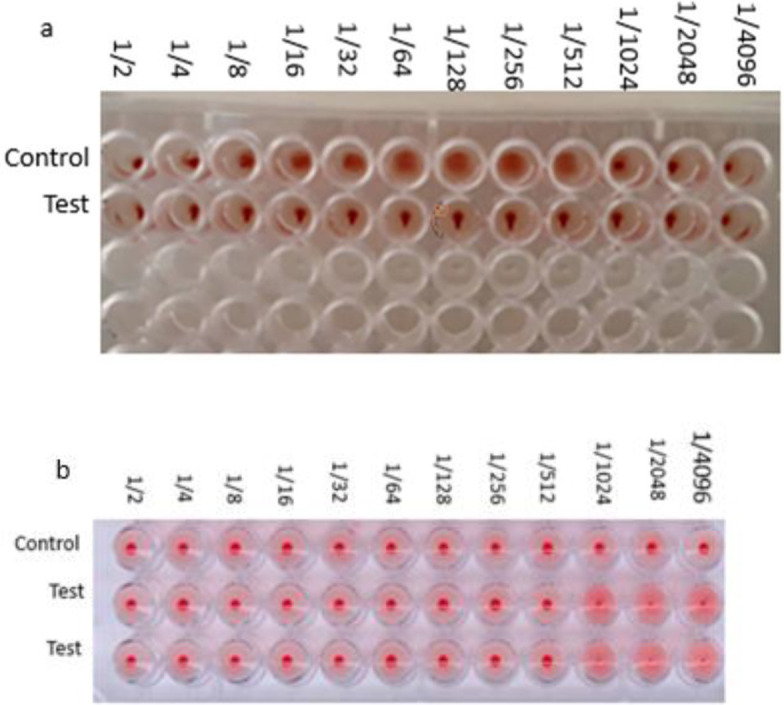
a. HA assay. a. HA: two-fold dilutions of influenza sample caused hemagglutination up to the 1:256 dilution; therefore the HA titer of this virus stock was 256. b. hemagglutination inhibition assay (HAI) of influenza viruses with chicken RBCs

The hemagglutination assay was performed on a 5 ml sample from virus-infected *Sf9* cell, and the observation of agglutination in the eighth well determined the antigen titer, which was equivalent to 256.1. This finding confirmed the bioactivity of HA in binding to its ligand on the surface of cells (Figure 5a).

The hemagglutination inhibition assay confirmed the neutralization activity of specific antibodies boosted after immunization. The analysis was guided with the use of the recombinant HA protein against serially-diluted reference anti-H1 antisera and 0.5% chicken RBCs (Figure 5b).

***Immune responses assessment***

**Figure 6:**
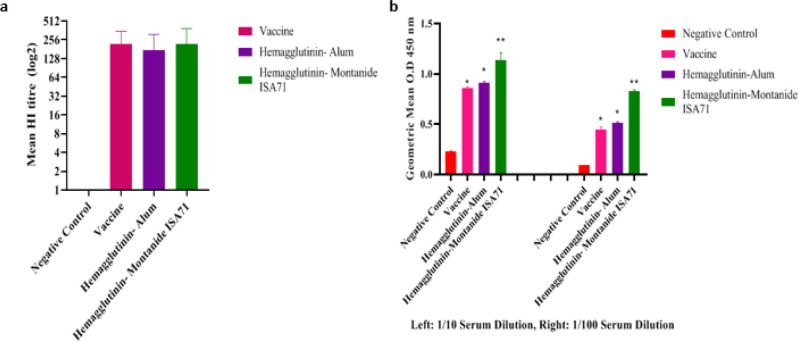
The results of ELISA and mean HI test for influenza viruse (H1N1). a: HI anti body titer (total IgG) boosted against 25 µl montanide+ rHA. b: the geometric mean determined by ELISA (between three vaccine groupsin two dilution (1:10 , 1:100)

Two weeks after the last injection, the induced immune response was assessed by HI and ELISA. The results showed that all groups were immunized with recombinant proteins (protein + alum; protein + Montanide ISA 71) and inactivated influenza viruses. The commercial vaccine significantly induced more antibodies against the A/California/07/2009(H1N1) strain, when compared with the control group (Figure 6a). The findings suggested that all immunized groups with recombinant protein (protein + Montanide ISA 71) and commercial vaccine significantly induced antibodies against the H1N1 negative control group. The vaccine formulation showed higher antibody responses, compared with the commercial vaccine (Figure 6b).

***Experimental challenge***

**Figure 7:**
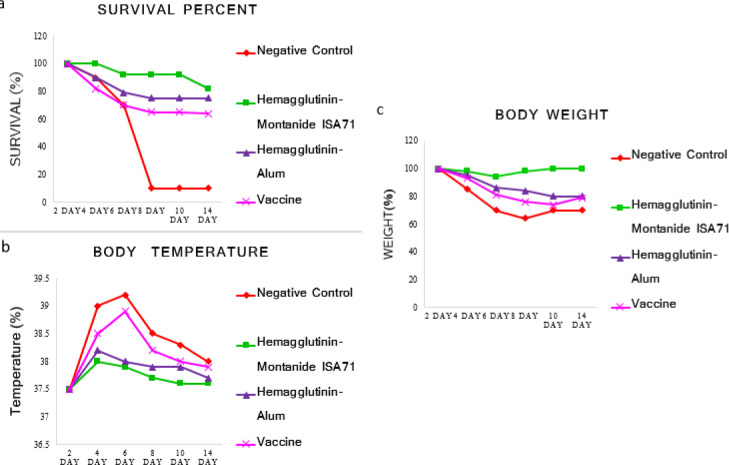
Challenges tests. The BALB/c mice was infected intranasally with influenza virus A/PR8/34(H1N1) and monitored for 2 weeks daily. a: survival percent, b: body temperature and c: body weight. In comparison with commercial vaccines and the negative control group, Hemagglutinin-Montanide ISA71 revealed less mortality rate, body temperature and weight change

The results of the challenge with the H1N1 virus showed that the hemagglutinin-Montanide ISA71 and the commercial vaccines were able to significantly protect the mice against the H1N1 virus when compared with the negative control group. The survival rate, weight changes, and body temperature in the vaccinated groups were lower than the control group. However, it seems that the protection rate in our novel new vaccine (96%) is higher than the commercial vaccine (80%) (Figure 7).

## Discussion

Although influenza infection is practically controlled through vaccination, research in the field of influenza vaccines is fast growing (25). Recombinant DNA technology and influenza genome sequencing have offered ample opportunities to build on novel approaches and can speed up vaccine development and production (26)e></EndNote>. Hemagglutinin is the main surface protein of the influenza virus, which is able to create a wide range of immune responses against the influenza virus and makes high titers of antibody that prevent virus attachment to the host cells (27).

In this study, eukaryotic expression vectors were applied. The findings demonstrated that the recombinant protein was expressed in an optimum manner. Hemagglutination of RBCs confirmed that this protein 3D structure is similar to the one on the virus surface, which can also enable attachment to its ligand on RBCs (22, 28). Recombinant proteins in the bare form are not immunogen, and their immunogenicity has to be improved (29). Many solutions, such as applying various cytokines or adjuvants, have been suggested (30). The expression system of baculovirus succeeded in producing multiple virus capsids, which is in line with the study of Baumart *et al*. (1998) who used the Bac-To-Bac system to produce HCV-like particles (VLPs) in insect cells (31). A 2006 study built on the baculovirus/insect cell system and it expressed two HA genes of the H5N2 strain (32). Furthermore, another research in 2010 developed the H1N1 influenza virus vaccine with a baculovirus system (33). In this study, a method devised in 1993 by Luckow *et al*. was applied to express recombinant hemagglutinin protein in insect cells (34). The findings of protein analysis by SDS-PAGE showed that transfecting insect cells with recombinant baculovirus with approximate MOI 10 and harvesting cells 72 hr after infection led to the production of recombinant proteins. 

Several pieces of research demonstrated that different insect cell lines are capable of expression and secretion of recombinant protein, of which two cell lines sf9 and sf21 are more recommended because other cells, such as High Five as a host of recombinant baculovirus are more sensitive due to production of more protease which leads to digestion of the produced protein. In a study, researchers used the High Five cell line to express and produce influenza VLPs. This cell line was able to secrete hemagglutinin and matrix 1 proteins, which were morphologically and immunologically very similar to influenza viruses (35). 

A study in 2012 compared A / California / 07/2009 recombinant hemagglutinin with H1N1 pandemic influenza recombinant hemagglutinin. The results showed that the sensitivity and immunological activity of the recombinant protein expressed in the baculovirus system were much higher than the hemagglutinin produced in the egg-based system (36).

Ebrahimi *et al*. also researched the M2 antigen in 2009. The findings suggest that antibodies produced against this protected antigen (M2) reduced the rate of virus replication and subsequent disease severity, indicating that the region remains highly protected. However, the half-life and immunogenicity of the specific M2 antibody was less than the specific hemagglutinin antibody. These results claimed that the combination of M2 and hemagglutinin increases the efficiency of the target universal vaccine (37).

Immunoassay results demonstrated that in comparison with the commercial vaccine and the negative control group, the immunization of experimental mice with the candidate vaccine could induce specific antibody responses against the H1N1 strain. Past research has revealed that alum adjuvant in the formulation of influenza vaccine barely enhances immunogenicity. However, induction of humoral immune responses and neutralizing antibodies did not entirely fit this particular vaccine as it demands further improvement with a more potent adjuvant (38). These results align with the previous studies suggesting that oil-based adjuvants can induce stronger immune responses and results in a higher protection rate (39, 40). 

After the immunogenicity study, the efficacy of vaccines is assessed by the viral challenge of experimental mice. The results of the challenge with mouse-adapted influenza A/PR8/34 (H1N1) virus showed that vaccination with the commercial vaccine and the vaccine candidate—developed in this study—could protect experimental mice, compared with the mice in the control group. In other words, the vaccinated mice survived as the vaccine protected them from increased body temperature and body weight loss. In the control group, the survival rate decreased dramatically, and body weight loss and severe fever occurred. These findings showed that antibodies, which developed during immunization, neutralized the virus and inhibited the virus pathogenesis in the vaccinated groups, while such changes were not observed in the control group. In addition, immunogenicity and efficacy studies revealed that our oil-based vaccine candidate was more potent than the commercial vaccine. Jafari *et al*. (2017) suggested that the formulation of the influenza vaccine in an oil-based adjuvant was more potent than an alum-based vaccine in induction of specific antibodies, which supported the findings of the present research (39). In conclusion, both the commercial vaccine and the candidate vaccine—which was expressed in the insect cell line and formulated in oil-based adjuvant—significantly boosted the immunogenicity and protectivity in the mouse model, highlighting the potentiality of the vaccine developed in this study.

## Conclusion

To sum up, the Bac-to-Bac baculovirus expression system efficiently produced HA protein of H1N1 in insect cells. Comparing adjuvanted antigen with the commercial vaccine against A/California/07/2009(H1N1) showed that long-lived immune response in mice was better induced with adjuvanted antigen. In addition, Montanide ISA 71 adjuvant practically was better than alum indicating the oil emulsion adjuvant in immunogenicity. Although a high immune response was induced in the early days after vaccination, long-live high antibody titer should be considered as an important factor. Therefore, choosing an appropriate adjuvant is essential in high immune response that is properly demonstrated in the present study.

## Authors’ Contributions

MM and MT Study conception and design; SZ Data analysis and draft manuscript preparation; SH, R JR, and MM Critical revision of the paper; MT Supervision of the research; SZ, MT, SH, RJ, MM Final approval of the version to be published.

## Conflicts of Interest

The authors declare no conflicts of interest. 
